# Supplementary material for: Content-rich biological network constructed by mining PubMed abstracts
Source: BMC Bioinformatics. 2004 Oct 8;5:147. doi: 10.1186/1471-2105-5-147 (PMC528731; doi:10.1186/1471-2105-5-147)
Supplement: Additional File 2 — The original results of the above study (non-essential files are deleted to keep the file size under the limit set by BMC bioinformatics). [file 1471-2105-5-147-S2.bz2 › chilibotAdditionalFile2/dip05/219ID7617032E728/html/IFNGR1_IFNG.html]

 


 **IFNGR1** and **IFNG** 
  
Found 72 abstracts in PubMed, retrieved 05.  
 

 What does Google say? 
 PDF only 
| .edu only 

---

**Interactive relationship** (e.g. stimulation, inhibition, etc)

**Non-interactive relationship** (e.g. studied together, co-existance, homology, etc.)

- We previously showed decreased OPN expression in the granulomas of patients with disseminated mycobacterial infection in the setting of mutation of the IFN gamma  [ **IFNG** ]  receptor 1 IFNGR 1  [ **IFNGR1** ] .  Ref: 12804068 J Interferon Cytokine Res, 2003
- Interferon gamma  [ **IFNG** ]  receptor 1  [ **IFNGR1** ]  IFNgammaR1 deficiency is a rare inherited immunodeficiency.  Ref: 12805054 Blood< MedlineTA>Blood, 2003
- Among the 12 down regulated genes, interferon gamma  [ **IFNG** ]  receptor 1  [ **IFNGR1** ]  was most notable x1 27.3.  Ref: 12823984 Exp Eye Res, 2003
- We identified a novel heterozygous single nucleotide substitution 1400 T right curved arrow C Leu 467 Pro in the seventh exon of the interferon gamma  [ **IFNG** ]  receptor 1  [ **IFNGR1** ]   **IFNGR1**  gene.  Ref: 12851715 Int J Mol Med, 2003
- Since interferon gamma  [ **IFNG** ]  is the main cytokine responsible for defence against leishmanial parasites, it was thought possible that effective antileishmanial drugs may up regulate  **IFNGR1** .  Ref: 12803856 Ann Trop Med Parasitol, 2003
- A novel single nucleotide substitution, Leu 467 Pro, in the interferon gamma  [ **IFNG** ]  receptor 1  [ **IFNGR1** ]  gene associated with allergic diseases.  Ref: 12851715 Int J Mol Med, 2003
- Persistent Mycobacterium avium infection following non myeloablative allogeneic peripheral blood stem cell transplantation for Interferon gamma  [ **IFNG** ]  receptor 1  [ **IFNGR1** ]  deficiency.  Ref: 12805054 Blood< MedlineTA>Blood, 2003
- Apparently for the first time, the peripheral blood monocytes of individuals with active visceral leishmaniasis VL have been found to show reduced expression of interferon gamma  [ **IFNG** ]  receptor 1  [ **IFNGR1** ]   **IFNGR1** .  Ref: 12803856 Ann Trop Med Parasitol, 2003
- Antileishmanial drugs cause up regulation of interferon gamma  [ **IFNG** ]  receptor 1  [ **IFNGR1** ] , not only in the monocytes of visceral leishmaniasis cases but also in cultured THP1 cells.  Ref: 12803856 Ann Trop Med Parasitol, 2003
